# YTHDF3 Promoted Gastric Cancer Progression by Facilitating m^6^A‐Dependent Degradation of APC mRNA

**DOI:** 10.1155/ancp/5045743

**Published:** 2026-09-15

**Authors:** Cao Zhang, Jun Wen, Zijian Liu, Ruyi Li, Yaqin He

**Affiliations:** ^1^ Department of Gastrointestinal Surgery, General Hospital of Ningxia Medical University, Yinchuan, China, nxmu.edu.cn; ^2^ The First Clinical College of Medicine, Ningxia Medical University, Yinchuan, China, nxmu.edu.cn; ^3^ Laboratory of Surgery, General Hospital of Ningxia Medical University, Yinchuan, China, nxmu.edu.cn

**Keywords:** APC, m^6^A, stomach adenocarcinoma, Wnt/β-catenin signaling, YTHDF3

## Abstract

**Background:**

Stomach adenocarcinoma (STAD) is a highly prevalent malignancy. YTH domain family protein 3 (YTHDF3), an m^6^A reader, correlates with worse survival outcomes in STAD. The aim was to study the impact of YTHDF3 on STAD cells and its underlying regulatory mechanisms.

**Methods:**

Epidemiologically, the GEPIA and Kaplan–Meier Plotter databases were used to examine YTHDF3 expression in STAD and to assess its association with prognosis, with poor prognosis defined by overall survival (OS). Clinical parameters were also analyzed to validate the prognostic value of YTHDF3. Experimentally, STAD patients were selected as the study objects. Immunofluorescence was used to confirm YTHDF3 expression in STAD tissues. In vitro assays examined YTHDF3/Adenomatous polyposis coli (APC) regulation of Wnt signaling.

**Results:**

Epidemiologically, YTHDF3 elevation in STAD (*p* < 0.05) predicted poorer OS (hazard ratio [HR] = 1.46, 95% confidence interval [95% CI]: 1.17–1.81, *p* = 0.00066). High YTHDF3 expression was also significantly associated with advanced T stage (*p* = 0.0069), N stage (*p* = 0.0088), and positive distant metastasis (*p* = 0.0076). Experimentally, elevated YTHDF3 expression was observed in STAD tissues and cells (all *p* < 0.001). Silencing YTHDF3 suppresses AGS malignancy and promotes apoptosis. Conversely, YTHDF3 overexpression aggravated the malignant phenotype of STAD (all *p* < 0.001). Mechanistically, silencing YTHDF3 promoted APC expression (*p* < 0.001) in an m^6^A‐dependent manner. Rescue experiments proved silencing APC reversed YTHDF3‐knockdown‐mediated repression of Wnt/β‐catenin signaling and AGS cell malignancy (all *p* < 0.001).

**Conclusion:**

YTHDF3 promoted STAD progression by regulating APC mRNA degradation.

## 1. Introduction

Stomach adenocarcinoma (STAD), one of the most prevalent malignant tumors globally, ranks fifth in incidence and third in mortality [1]. At present, STAD is mainly treated by surgical resection combined with postoperative chemotherapy [2]. However, despite improvements in surgical and adjuvant treatments, about 40% of patients suffering from STAD will advance to recurrence following surgery [3], and in these patients with recurrence, the overall survival (OS) rate over a 5‐year period is below 30% [4]. In patients with STAD, tumor metastasis significantly influences the negative prognosis, so understanding the metastasis mechanism of STAD is of critical importance for its treatment.

Some studies have confirmed that epigenetic changes in tumor cells are an important way to acquire the ability of metastasis, and the regulation of N^6^‐methyladenine (m^6^A) modification sites in messenger RNA transcripts is one of the important reasons for epigenetic changes [5]. m^6^A is the earliest and most prevalent RNA modification identified in eukaryotes [6]. m^6^A modification plays a role in many aspects of gene expression regulation, such as RNA cleavage, maturation, nucleation, translation, and degradation, thereby affecting a range of physiological and pathological mechanisms, including embryonic development, maintenance of metabolic homeostasis, and tumorigenesis and development [7]. The homeostasis of m^6^A modification is controlled by three types of regulators: writer, which mainly refers to the m^6^A methyltransferase complex, mainly consisting of METTL3, METTL14, and Wilms tumor‐1‐associated protein (WTAP); eraser, which mainly refers to the m^6^A‐demethylase complex, including FTO and ALKBH5; and reader, which mainly composed of YTHDF1‐3, YTHDC1/2, and lGF2BP1/2/3 [8]. Several m^6^A regulators, including METTL3 [9], FTO [10], and YTHDF2 [11], have been identified to promote malignant progression in STAD. YTH domain family protein 3 (YTHDF3) is an m^6^A reader characterized by the vertebrate YTH domain, which regulates RNA fate by controlling the rate of RNA methylation. As a crucial reader of m^6^A modification, YTHDF3 modulates targets posttranscriptionally [12]. YTHDF3 has emerged as a critical factor in the advancement of pancreatic cancer [13], clear‐cell renal cell carcinoma [14], prostate cancer [15], and even thyroid cancer [16]. Moreover, YTHDF3 has been shown to be associated with the clinical prognosis of STAD [17]. Existing results have demonstrated that YTHDF3 promoted the progression of gastric cancer cells, including the STAD cell line MKN74 and the undifferentiated cell line HGC‐27, via the Wnt/β‐catenin pathway [18]. Given that HGC‐27 cells are derived from the metastatic lymph node of gastric cancer [19], this observation suggests that YTHDF3 may interfere with the Wnt/β‐catenin pathway, thereby potentially promoting more frequent lymph node metastases and contributing to poor clinical prognosis. However, its specific effects and regulatory mechanisms in STAD cells remain to be further explored.

Adenomatous polyposis coli (APC) is a vital tumor suppressor gene [20] and commonly mutated in human cancers [21]. Somatic mutations in APC occur in ~80% of sporadic colorectal carcinomas [22]. Functionally, APC is a member of the Wnt signaling pathway and a critical component of the β‐catenin destruction machinery [23]. It promotes β‐catenin phosphorylation and degradation, keeping cytoplasmic levels low without Wnt [24]. Consequently, the impairment of APC function resulted in abnormal nuclear accumulation of β‐catenin [25]. According to relevant reports, YTHDF2 in the YTHDF family could promote the degradation of m^6^A‐modified APC mRNA, thus participating in the progression of esophageal cancer [26]. Whether APC is a direct m^6^A target of YTHDF3 in STAD remains unclear.

Bioinformatics analysis and functional assays were performed on STAD cells with YTHDF3 knockdown and overexpression. The possible mechanism of YTHDF3 was investigated by RNA pull‐down and actinomycin D assays. This may provide a new perspective for understanding m^6^A modifications in STAD.

## 2. Methods

### 2.1. GEPIA and Kaplan–Meier Plotter Database Analysis

The GEPIA database (last accessed March 1st, 2024) [27] is a visual cancer big data analysis platform based on two well‐known transcriptome databases, The Cancer Genome Atlas (TCGA) and Genotype‐Tissue Expression (GTEx) Project, which was used to verify the expression level of YTHDF3 in STAD. The Kaplan–Meier plotter database (last accessed March 1st, 2024) is a widely used online tool for tumor prognosis, and we used it to analyze the prognostic value of YTHDF3 in STAD. Poor prognosis was defined based on OS as the primary endpoint. Patients were grouped by the median YTHDF3. To further validate the prognosis, associations between YTHDF3 expression and clinical parameters (T stage: T1–T2 vs. T3–T4; N stage: N0 vs. N1–N3; and distant metastasis: negative vs. positive) were assessed using the chi‐square test.

### 2.2. STAD Tumor Samples

STAD tissue and matched paracancerous tissue were obtained from 72 STAD patients [27] who underwent total or partial gastrectomy and were admitted to the General Hospital of Ningxia Medical University from March 2022 to September 2024. Eligible patients meeting the criteria were consecutively enrolled. The study’s recruitment period spanned roughly 3 years to reach the predefined sample size. Sample size (*n* = 72) was calculated by G ^∗^Power 3.1.9.7 for a two‐tailed paired *t*‐test (*α* = 0.05, power = 0.80). The calculation was based on an expected mean difference of 0.39 and a standard deviation of 1.0 for the paired difference in YTHDF3 expression (ΔCt or relative expression) between STAD and adjacent normal tissues, derived from pilot data. The goal of the epidemiological analysis was to characterize YTHDF3 expression in STAD clinical samples, specifically comparing its levels between tumor tissues and adjacent normal tissues. STAD patients were enrolled per criteria [27]: Inclusion criteria: (1) adults; (2) histologically confirmed gastric adenocarcinoma; (3) no preoperative antitumor treatment, including radiotherapy, chemotherapy, or immunotherapy. Exclusion criteria: (1) other malignancies; (2) active connective tissue disease; and (3) patients who had received any adjuvant treatment (as defined above) prior to sample collection. Surgical techniques were performed according to the Chinese Society of Clinical Oncology (CSCO) Gastric Cancer Guidelines. This study was approved by the Ethics Committee of the General Hospital of Ningxia Medical University (Approval Number KYLL‐2023‐0096), and all participants signed informed consent forms. To define high and low YTHDF3 expression levels in the 72 STAD patients, the following quantitative criteria were used. Based on the quantitative reverse transcription polymerase chain reaction (qRT‐PCR) results, we classified the patients with a higher expression level than the median value into the high expression group and the patients with a lower expression value than the median value into the low expression group.

### 2.3. Immunofluorescence

Immunofluorescence was conducted utilizing an anti‐YTHDF3 antibody (1:50, ab290734). After the sections were dewaxed and hydrated, they were closed with a 5% BSA solution for 1 h. The next step involved adding the primary antibody and incubating it overnight at 4°C. The following day, a secondary antibody (1:2000, ab6789) was applied and incubated for 1 h. Finally, the sections were examined using a fluorescence microscope.

### 2.4. Cell Culture

GES‐1, HGC‐27, NCI‐N87, SNU‐16, and AGS cells were obtained from the CAS cell bank (Shanghai) [27]. All cells were maintained with 5% CO_2_ and 95% air at 37°C. In addition, SNU‐16 cells received treatment with 3‐deazaadenosine (DAA) (50 μM) (an inhibitor of m^6^A methylation) (Cayman, USA) to elucidate the role of m^6^A modification.

### 2.5. Cell Transfection

Short interfering RNAs (siRNAs) designed to target YTHDF3 and APC, along with their corresponding negative controls (si‐NC), were obtained from Genechem (Shanghai, China). Plasmids overexpressing YTHDF3 (YTHDF3) and the corresponding control (NC) were cloned into the pcDNA3.1 vector (Genechem, China). Lipofectamine 3000 was used for the transfection of the overexpressed plasmid and siRNA of the indicator gene. The target sequences for siRNAs are shown as follows:

si‐YTHDF3‐1: 5′‐GGUGGAUUUCACCAGUUAAUG‐3′

si‐YTHDF3‐2: 5′‐AGAUGGUGUAUUUAGUCAACC‐3′

si‐YTHDF3‐3: 5′‐GGGCAAAUUUGAAGUUAAAUG‐3′

si‐APC‐1: 5′‐CACCAUGUGGAUCAGCCUA‐3′

si‐APC‐2: 5′‐CAGAGGAAGGAGAUAUUC‐3′

si‐APC‐3: 5′‐GGATCAGCCTATTGATTAT‐3′

si‐NC: 5′‐TTCTCCGAACGTGTCACGA‐3′.

### 2.6. CCK‐8 Assay

AGS cells, either transfected with si‐YTHDF3 or not, along with SNU‐16 cells transfected with or without the YTHDF3 plasmid, were plated into 96‐well plates according to the density of 2 × 10^3^ cells/well and cultivated over the course of days 1, 2, 3, 4, and 5, respectively. A volume of 10 μL of CCK‐8 reagent, provided by Beyotime Biotechnology (Shanghai, China), was added to the samples and incubated at 37°C for 2 h, and then the absorbance at 450 nm was measured using a microplate reader (BioTek Instruments, Winooski, VT, USA) and plotted.

### 2.7. EdU Assay

Logarithmically growing AGS and SNU‐16 cells were seeded into 96‐well plates at a density of 4 × 10^3^ cells/well, and each well received 100 μL of 50 μM EdU medium (RiboBio, Guangzhou, China). Fixation was performed using 4% paraformaldehyde. After discarding the fixative, permeabilization of the cells was carried out with 0.5% Triton X‐100 for 10 min, followed by staining in the staining solution for 30 min. All images were captured using an optical microscope.

### 2.8. Cell Invasion Assays

A diluted Matrigel solution (60 μL) was added to the polycarbonate membrane in the Transwell chamber, and log‐phase cells were resuspended in serum‐free medium at 1 × 10^4^ cells/mL. Prior to seeding, cells were treated with 1.8 mmol/L hydroxyurea (Sigma–Aldrich, Merck KGaA) for 12 h to inhibit cell proliferation. Cells were seeded in the upper chamber with a FBS‐containing medium in the lower chamber. After 24 h, the invaded cells on the lower membrane were fixed and stained with crystal violet. The number of cells that penetrated the membrane was counted using an inverted microscope (TE2000, Nikon, Tokyo, Japan), and the count of cells that penetrated the membrane reflected their invasive potential.

### 2.9. TUNEL Staining

Both transfected and nontransfected AGS and SNU‐16 cell suspensions were plated onto 24‐well plates with coverslips, fixed with 4% paraformaldehyde for 30 min, permeabilized with 0.5% Triton X‐100 on ice for 10 min, and washed again with PBS for 5 min. A volume of 100 μL of TUNEL detection solution (In Situ Cell Death Detection Kit, Roche) was dispensed into each well. Following this, the cells were mounted with an antifade reagent.

### 2.10. Flow Cytometry Analysis of Apoptosis

Apoptosis was assessed using an Annexin V‐FITC/propidium iodide (PI) staining (Annexin V‐FITCApoptosis Detection Kit; BD Biosciences) according to the manufacturer’s instructions. Briefly, Cells were suspended in binding buffer (1 × 10^6^/mL), stained with 5 μL Annexin V‐FITC/PI, and analyzed by flow cytometry (BD FACSCalibur, USA). Data were analyzed using Flowjo V10 software (Tree Star, San Francisco, CA, USA).

### 2.11. RNA Stability Assay

At the logarithmic growth stage, AGS and SNU‐16 cells were exposed to actinomycin D (2 mg/mL, Sigma, A4262, USA). Total RNA was extracted from each group at 0, 60, 120, 180, and 240 min, respectively. The expression levels of APC were quantified via qRT‐PCR, and a decay curve for APC was constructed accordingly.

### 2.12. RNA Pull‐Down Assays

Biotinylation of RNA was accomplished using the Pierce RNA 3′‐end Biotinylation Kit (Thermo Fisher Scientific, Waltham, MA, USA). The protein complex was bound with the operation instructions of the RNA pull‐down kit (Pierce, IL, USA), the protein complex was adsorbed with magnetic beads, and the magnetic beads were washed. After the test, the YTHDF3 antibody (1:800, ab220161) was applied to Western blot.

### 2.13. Dual‐Luciferase Reporter Assay

HEK‐293T cells were cotransfected with wild‐type (Wt) or mutant (Mut) APC 3′‐UTR luciferase reporter plasmids (pMIR‐REPORT), in which the Wt construct contains the 3′ UTR sequence of the APC‐208 transcript (ENST00000508624) harboring a predicted m^6^A modification site (genomic coordinates: chr5:112837919–112838021), and the Mut construct carries point mutations disrupting this site, together with either si‐NC or si‐YTHDF3. After 48 h of transfection, dual‐luciferase activity was measured using the Dual‐Luciferase Reporter Assay System (Promega, Madison, WI, USA). Firefly luciferase activity was normalized to Renilla luciferase activity to correct for the transfection efficiency.

### 2.14. TOP/FOP‐Flash Luciferase Reporter Assay

Wnt/β‐catenin signaling activity was evaluated using the TOP/FOP‐Flash luciferase reporter assay. Cells were cotransfected with TOP flash or FOP flash expression plasmid (Beyotime, Shanghai, China) using Lipofectamine 3000. At 48 h posttransfection, Firefly and Renilla luciferase activities were measured using the Dual‐Luciferase Reporter Assay System (Promega, Madison, WI, USA). Firefly luciferase activity was normalized to Renilla luciferase activity, and Wnt pathway activity was expressed as the normalized TOP/FOP ratio. TOP/FOP ratio indicates Wnt/β‐catenin activity.

### 2.15. 3‐[4,5‐Dimethylthiazol‐2‐yl]‐2,5‐Dephenyl Tetrazolium Bromide (MTT) Assay

The cytotoxicity of DAA was determined by the MTT assay. AGS and SNU‐16 cells were seeded at 2 × 10^4^ cells/mL in 2% FBS‐DMEM. Upon attachment, media with different concentrations of DAA (25, 50, 75, and 100 μM) were substituted instead. After culturing the cells in DAA for 2 days, cells were incubated with 5 mg/mL MTT in serum‐free DMEM for 4 h, then formazan was dissolved in DMSO, and absorbance was read at 590 nm (Bioteke, Beijing) [27].

### 2.16. qRT‐PCR

Total RNA was isolated from cells and tissues using the Trizol reagent (Invitrogen, Thermo Fisher Scientific, Waltham, MA, USA). RNA was reverse‐transcribed (Applied Biosystems) and amplified by qRT‐PCR (Vazyme SYBR). YTHDF3 and APC mRNA levels were normalized to GAPDH and calculated by 2^−△△CT^. Three repetitions of the experiment were conducted. Primer sequences are as follows:

YTHDF3 forward, (5′‐3′): 5′‐TGTTGTGGACTATAATGCGTATGC‐3′ and reverse, (5′‐3′), 5′‐AAGCGAATATGCCGTAATTGGTTA‐3′;

APC forward, (5′‐3′): TTGTTTTTTTGTGTTGTAAAAATTATAGTA and reverse, (5′‐3′), ACACCTCCATTCTATCTCCAATAAC.

c‐MYC forward, (5′‐3′): TTTTCGGGTAGTGGAAAACCAGC and reverse, (5′‐3′), AGTAGAAATACGGCTGCACCGAC;

Cyclin D1 forward, (5′‐3′): GGATGCTGGAGGTCTGCGA and reverse, (5′‐3′), TAGAGGCCACGAACATGCAAGT;

GAPDH forward, (5′‐3′): 5′‐TGACTTCAACAGCGACACCCA‐3′ and reverse, (5′‐3′), 5′‐CACCCTGTTGCTGTAGCCAAA‐3′. The mRNA levels of c‐MYC and Cyclin D1 were quantified as downstream targets of the Wnt/β‐catenin pathway. Downregulation of these genes indicates reduced Wnt/β‐catenin signaling activity.

### 2.17. Western Blot

RIPA lysis buffer (Beyotime, P0013B, Shanghai, China) containing protease inhibitors and phosphorylase inhibitors was used to isolate total cellular proteins. Nuclear proteins were extracted using the nuclear protein extraction kit (Solarbio, Beijing, China) in accordance with the provided protocol and subsequently stored at –80°C. For Western blot, proteins (10 μg) were resolved by SDS‐PAGE, transferred to PVDF (Millipore), blocked with 5% BSA for 2 h, and probed with primary antibodies overnight at 4°C. Secondary antibody treatment followed, with incubation at room temperature for 1.5 h. Primary antibodies were as follows: YTHDF3 (1:800, ab220161), APC (1:200, ab40778), total β‐catenin (*t*‐β‐catenin) (1:2000, ab32572), nuclear β‐catenin (*N*‐β‐catenin) (1:2000, ab32572), phosphorylated β‐catenin (*p*‐β‐catenin,1:10000, ab81305), GAPDH (1:1000, ab8245), and H3 (1:10000, ab1791). The following parameters were defined: *t*‐β‐catenin represents the total cellular β‐catenin protein level; *N*‐β‐catenin refers to the nuclear‐localized fraction of β‐catenin, which serves as a marker for active Wnt signaling; and *p*‐β‐catenin refers to the phosphorylated form of β‐catenin, which is targeted for proteasomal degradation and thus considered inactive.

### 2.18. Statistical Analysis

Data are the mean ± SD. Between‐group differences (STAD vs. normal) were analyzed by the Student’s *t*‐test using SPSS 22.0 (IBM, NY). Group differences were analyzed by one‐way ANOVA (LSD post hoc), with *p* < 0.05 considered significant.

## 3. Results

### 3.1. YTHDF3 Overexpression Correlates With Poor STAD Prognosis

**Figure 1:**
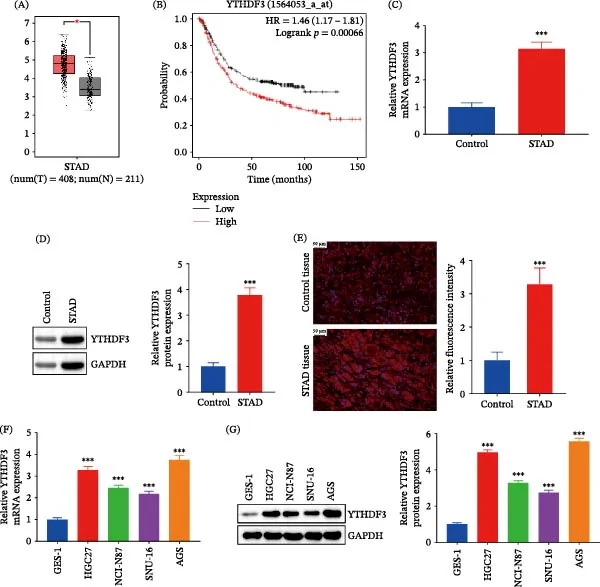
YTHDF3 is overexpressed in stomach adenocarcinoma (STAD) and is associated with poor prognosis of STAD. (A) The GEPIA database was used to analyze the expression of YTHDF3 in STAD tissues. (B) Survival analysis was conducted using Kaplan–Meier plotter. (C) The expression of YTHDF3 in STAD tissues and paracancerous tissues was detected through quantitative reverse transcription polymerase chain reaction (qRT‐PCR, *n* = 72). (D) Western blot was performed to detect the expression of YTHDF3 in STAD tissues and paracancerous tissues. (E) Immunofluorescence was employed to determine the expression of the YTHDF3 in STAD tissues. Scale bars indicate 50 μm. (F) qRT‐PCR analysis of mRNA levels of YTHDF3 in gastric epithelial cells (GES‐1 cells) and STAD cell lines. (G) The protein of YTHDF3 in gastric epithelial cells (GES‐1 cells) and STAD cell lines was detected by Western blot.  ^∗∗∗^ *p* < 0.001 vs. control or GES‐1 group.

Utilizing the GEPIA database, we evaluated the expression of YTHDF3 in STAD. Our findings revealed that YTHDF3 was elevated in STAD (*p* < 0.05, Figure 1A) and predicted poor survival (HR = 1.46, *p* = 0.00066, Figure 1B). Based on the database analysis results, STAD and paired normal tissues (*n* = 72) were collected. The cohort included 48 males and 24 females, with a mean age of 66 years and an age range spanning from 51 to 81 years. 30 cases with tumor size <5.0 cm and 42 cases with tumor size ≥5.0 cm were included; for T stage, there were 21 T1–T2 and 51 T3–T4 stage cases, and for N stage, there were 23 N0 and 49 N1–N3 stage cases. We then found abnormally high YTHDF3 expression in STAD tissues compared to paracancerous tissues (*p* < 0.001, Figure 1C, D). Immunofluorescence staining further validated increased YTHDF3 expression in STAD tissues relative to paracancerous tissues (*p* < 0.001, Figure 1E). Next, we found STAD cell lines showed higher YTHDF3 levels (*p* < 0.001, Figure 1F, G). In addition, dividing STAD patients based on the median YTHDF3 expression identified distinct subgroups. Comparison of clinical features revealed a strong association between high YTHDF3 expression and advanced tumor stages (T and N) (*p* = 0.0069 and 0.0088) and distant metastasis (*p* = 0.0076) (Table 1). Altogether, high YTHDF3 predicts poor STAD survival.

**Table 1:** Associations between YTHDF3 protein expression and clinicopathological characteristics in STAD patients (*n* = 72).

Parameters	Category	Number	Expression of YTHDF3		*p*‐Value
			High (35)	Low (37)	
Age	<60	26	11	15	0.4211
	≥60	46	24	22	
Gender	Male	48	24	24	0.7388
	Female	24	11	13	
Tumor site	<5 cm	30	13	17	0.4489
	≥5 cm	42	22	20	
T stage	T1–T2	21	5	16	0.0069
	T3–T4	51	30	21	
N stage	N0	23	6	17	0.0088
	N1–N3	49	29	20	
Distant metastasis	Negative	48	18	30	0.0076
	Positive	24	17	7	

*Note:* The *p*‐value was measured by the Chi‐square test. *p* < 0.05 indicates statistical significance.

### 3.2. Silencing YTHDF3 Inhibits AGS Malignancy and Triggers Apoptosis

**Figure 2:**
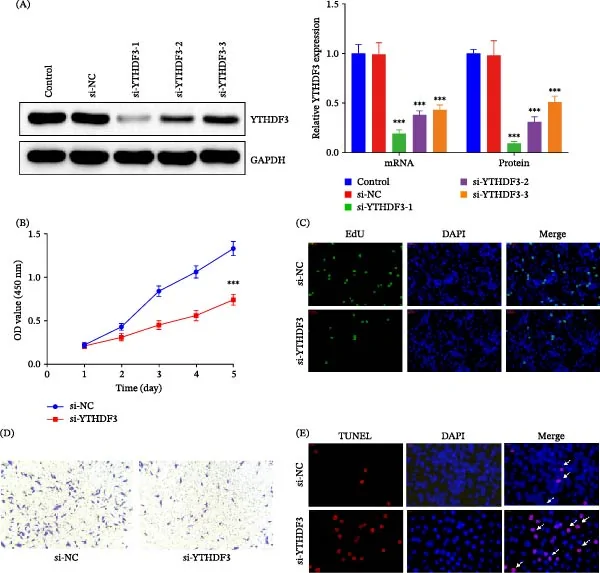
Silencing YTHDF3 inhibits the proliferation and invasion and promotes apoptosis in AGS cells. (A) Levels of YTHDF3 in AGS cells transfected with the three short interfering RNAs (siRNAs) (si‐YTHDF3‐1, si‐YTHDF3‐2, and si‐YTHDF3‐3) were analyzed by quantitative reverse transcription polymerase chain reaction (qRT‐PCR) and Western blot. (B) Cell counting kit‐8 (CCK‐8) assay was performed to detect activity of AGS cells after YTHDF3 silencing. (C) The proliferation of AGS cells after YTHDF3 silencing was examined by the 5‐ethynyl‐2′‐deoxyuridine (EdU) assay. (D) The invasion of AGS cells after YTHDF3 silencing was verified by transwell assay. (E) Terminal deoxynucleotidyl transferase‐mediated dUTP nick end labeling (TUNEL) staining was used to detect apoptosis of AGS cells in each group.  ^∗∗∗^ *p* < 0.001 vs. si‐NC group.

Results in Figure 1F,G showed that YTHDF3 expression was highest in AGS cells among STAD cell lines. To investigate the impact of YTHDF3 on STAD cellular phenotypes, we silenced YTHDF3 in AGS cells through siRNA transfection. Three siRNAs (si‐YTHDF3‐1/2/3) were designed, and the knockdown efficiency was validated. The findings indicated that si‐YTHDF3‐1 exhibited a greater silencing effect than the others, so it was used in the subsequent experiments (*p* < 0.001, Figure 2A). Then, through CCK‐8 assay, it was found that YTHDF3 silencing reduced STAD cell viability (*p* < 0.001, Figure 2B), and silencing YTHDF3 significantly reduced the proliferation ability of AGS cells (Figure 2C). The transwell assay also showed that silencing YTHDF3 reduced AGS invasion (Figure 2D), and TUNEL assay results also showed that silencing YTHDF3 in AGS cells resulted in an increased apoptosis rate (Figure 2E). Briefly speaking, the results suggested that silencing YTHDF3 inhibited AGS cell malignancy.

### 3.3. Overexpression of YTHDF3 Promotes the Proliferation and Invasion and Inhibits Apoptosis in SNU‐16 Cells

**Figure 3:**
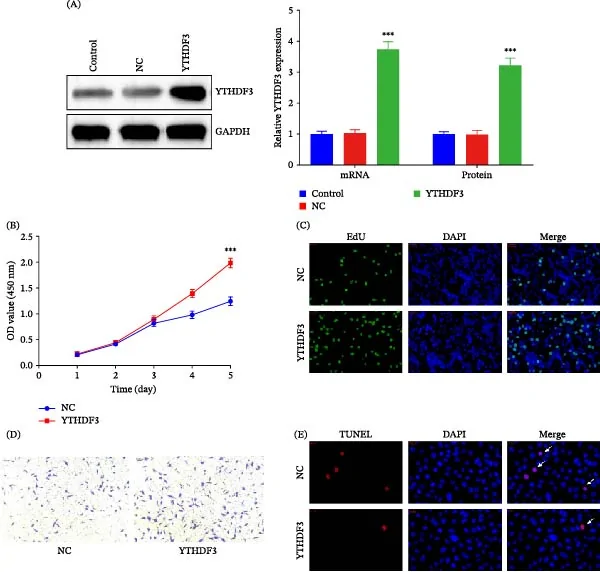
Overexpression of YTHDF3 promotes the proliferation and invasion and inhibits apoptosis in SNU‐16 cells. (A) Levels of YTHDF3 in SNU‐16 cells transfected with the YTHDF3 overexpression plasmid were analyzed by quantitative reverse transcription polymerase chain reaction (qRT‐PCR) and Western blot. (B) Cell counting kit‐8 (CCK‐8) assay was performed to detect activity of SNU‐16 cells after YTHDF3 overexpression. (C) The proliferation of SNU‐16 cells after YTHDF3 overexpression was examined by the 5‐ethynyl‐2′‐deoxyuridine (EdU) assay. Nuclei were stained with DAPI (blue), and proliferating cells were labeled with EdU (green). (D) The invasion of SNU‐16 cells after YTHDF3 overexpression was verified by transwell assay. (E) Terminal deoxynucleotidyl transferase‐mediated dUTP nick end labeling (TUNEL) staining was used to detect apoptosis of SNU‐16 cells in each group.  ^∗∗∗^ *p* < 0.001 vs. NC group.

Based on the findings from Figure 1F,G, which indicated that YTHDF3 expression was lowest in SNU‐16 cells among STAD cell lines, we proceeded to introduce an overexpression plasmid encoding YTHDF3 into SNU‐16 cells. Successful transfection was proved by both qRT‐PCR and Western blot (*p* < 0.001, Figure 3A). Subsequent assays revealed that YTHDF3 overexpression significantly promoted the viability and proliferation of SNU‐16 cells (*p* < 0.001, Figure 3B, C). The transwell assay also confirmed that YTHDF3 exacerbated the invasion of SNU‐16 cells (Figure 3D). In addition, TUNEL staining showed that YTHDF3 could inhibit the apoptosis of SNU‐16 cells (Figure 3E). In conclusion, we concluded that the overexpression of YTHDF3 promoted malignancy of SNU‐16 cells.

### 3.4. YTHDF3 Suppresses APC Expression by Promoting m^6^A‐Dependent Degradation of APC mRNA

**Figure 4:**
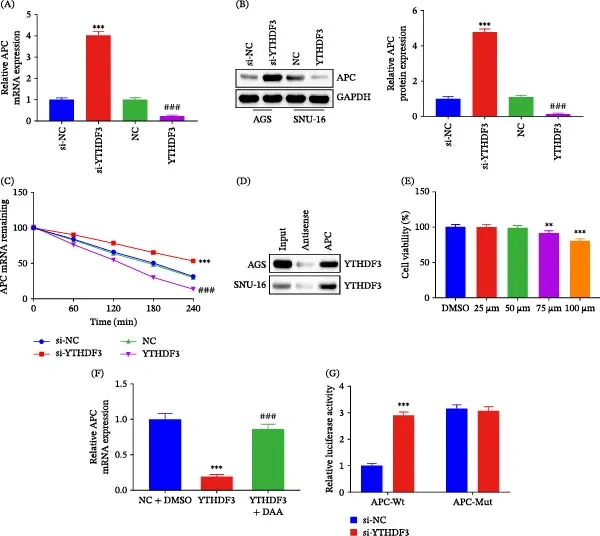
YTHDF3 suppresses APC expression by promoting m^6^A‐dependent degradation of APC mRNA. (A) Levels of APC in AGS cells transferred to si‐YTHDF3 and SNU‐16 cells transferred to YTHDF3 overexpressing plasmid were analyzed by quantitative reverse transcription polymerase chain reaction (qRT‐PCR). (B) Western blot was performed to detect APC protein levels of AGS and SNU‐16 cells transfected as above. (C) The stability of APC mRNA was detected by actinomycin D. (D) An RNA pull‐down assay was used to detect the interaction between YTHDF3 and APC mRNA in AGS and SNU‐16 cells. (E) The cytotoxicity of 3‐deazaadenosine (DAA) was verified by MTT assay. (F) The mRNA level of APC was detected in SNU‐16 cells transfected with the YTHDF3 overexpressing plasmid with or without DAA treatment using qRT‐PCR. (G) HEK‐293T cells were cotransfected with wild‐type (Wt) or mutated (Mut) APC 3′‐UTR reporter vectors and si‐NC or si‐YTHDF3, followed by dual‐luciferase reporter assay.  ^∗∗^ *p* < 0.01,  ^∗∗∗^ *p* < 0.001 vs. si‐NC or DMSO group.  ^###^ *p* < 0.001 vs. NC or YTHDF3 group.

YTHDF2, a component of the YTHDF family in the m^6^A reader, could induce APC degradation by recognizing m^6^A‐modified APC mRNA, a commonly mutated tumor suppressor gene in cancer [26]. Given that YTHDF3, another member of YTHDF, also acted as an m^6^A reader and could bind to the m^6^A site to regulate the stability of target mRNA and protein translation [28], we conducted further research on the relationship between YTHDF3 and the m^6^A modification of APC. qRT‐PCR and Western blot assays revealed that silencing YTHDF3 upregulated APC expression, whereas overexpression of YTHDF3 downregulated APC expression (*p* < 0.001, Figure 4A, B). We further investigated whether YTHDF3 affected the stability of the APC mRNA. An RNA stability experiment indicated that the degradation rate of APC mRNA in AGS cells treated with actinomycin D in the si‐YTHDF3 group was notably slower than that in the si‐NC group at a specified time, and the degradation rate in SNU‐16 cells of the YTHDF3 group was significantly accelerated (*p* < 0.001, Figure 4C). Next, the RNA pull‐down assay showed that YTHDF3 could directly interact with APC (Figure 4D). In order to further explore whether APC mRNA was modified by YTHDF3‐mediated m^6^A, DAA was used to treat SNU‐16 cells as an m^6^A methylation inhibitor. To determine the experimental concentration of DAA, we initially treated SNU‐16 cells with various concentrations of DAA. The MTT assay showed that DAA concentrations below 50 μM were not cytotoxic. However, when DAA concentration was 75–100 μM, SNU‐16 cell viability was inhibited and cytotoxicity was generated (*p* < 0.01, *p* < 0.001, Figure 4E). Therefore, we chose DAA with a concentration of 50 μM as the experimental dose concentration. qRT‐PCR showed that compared with NC + DMSO, the mRNA level of APC in the YTHDF3 group was significantly decreased, while DAA blocked the degradation of APC by YTHDF3. In addition, YTHDF3‐knockdown‐enhanced WT (m^6^A‐containing) APC 3′UTR luciferase activity, whereas the Mut was active and resistant to this effect (*p* < 0.001, Figure 4G). Collectively, YTHDF3 suppressed APC via m^6^A‐mediated mRNA degradation.

### 3.5. Silencing APC Reverses the Inhibition of Wnt/β‐Catenin Signaling Induced by Silencing YTHDF3

**Figure 5:**
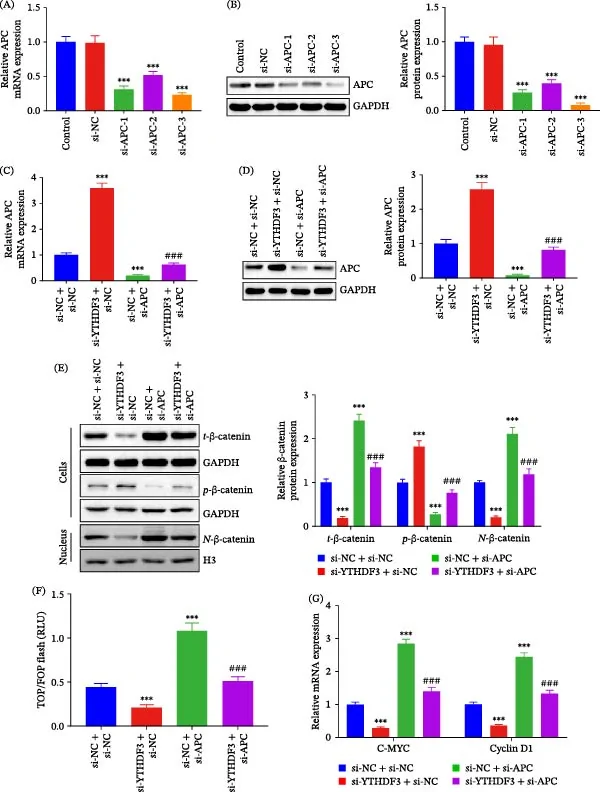
Silencing APC reverses the inhibition of Wnt/β‐catenin signaling induced by silencing YTHDF3. (A) Levels of APC in AGS cells transfected with the three short interfering RNAs (siRNAs) (si‐APC‐1, si‐APC‐2, and si‐APC‐3) were analyzed by quantitative reverse transcription polymerase chain reaction (qRT‐PCR). (B) Western blot analysis of levels of APC in AGS cells transfected with the three siRNAs. (C) qRT‐PCR analysis of levels of APC in AGS cells in different transfection groups: si‐NC + si‐NC, si‐YTHDF3 + si‐NC, si‐NC + si‐APC, or si‐YTHDF3 + si‐APC. (D) Western blot analysis of levels of APC in AGS cells in different transfection groups. (E) Western blot was performed to detect the protein levels of total β‐catenin (*t*‐β‐catenin), nuclear β‐catenin (*N*‐β‐catenin), and phosphorylated β‐catenin (*p*‐β‐catenin) in different groups of AGS cells. (F) The activity of β‐catenin was detected through TOP/FOP‐Flash luciferase reporter assay. (G) qRT‐PCR analysis of mRNA levels of c‐MYC and Cyclin D1.  ^∗∗∗^ *p* < 0.001 vs. si‐NC or si‐NC + si‐NC.  ^###^ *p* < 0.001 vs. si‐YTHDF3 + si‐NC.

Next, we first used three independent siRNAs to knock down APC in AGS cells. qRT‐PCR and Western blot analyses showed si‐APC‐1 had a higher silencing effect than the others, and therefore it was used for the following experiments (*p* < 0.001, Figure 5A, B). Subsequently, si‐APC and si‐YTHDF3 were cotransferred to AGS cells, and the results showed that silencing YTHDF3 could promote the expression of APC, while cotransferred with si‐APC partially reversed this promoting effect (*p* < 0.001, Figure 5C, D). Since β‐catenin is the downstream mediator of APC [29], we then explored the effects of YTHDF3 and APC on β‐catenin. The Western blot assay showed that silencing YTHDF3 significantly decreased the levels of both *t*‐β‐catenin and *N*‐β‐catenin while increasing the level of *p*‐β‐catenin. However, cotransfection with si‐APC partially reversed these effect (*p* < 0.001, Figure 5E). TOP/FOP‐Flash luciferase reporter assay revealed that silencing YTHDF3 significantly reduced β‐catenin transcriptional activity, which was partially restored by cotransfection with si‐APC (*p* < 0.001, Figure 5F). The mRNA levels of downstream target genes of the Wnt/β‐catenin signaling pathway, including c‐MYC and Cyclin D1, were markedly downregulated upon YTHDF3 silencing, which was attenuated by cotransfection with si‐APC (*p* < 0.001, Figure 5G). To sum up, the silencing of APC reversed the YTHDF3 silencing‐induced the inhibition of Wnt/β‐catenin signaling.

### 3.6. Silencing APC Reversed the Promoting Effect of Silencing YTHDF3 on AGS Cells

Finally, we determined whether YTHDF3 regulates the malignancy of STAD cells by modulating the APC expression. AGS cells underwent transfection with combinations of si‐NC + si‐NC, si‐YTHDF3 + si‐NC, si‐NC + si‐APC, or si‐YTHDF3 + si‐APC, respectively. We found YTHDF3‐knockdown‐inhibited cell viability and proliferation, which was reversed by si‐APC (*p* < 0.001, Figure 6A, B). Similarly, silencing YTHDF3 inhibited the invasion of AGS cells, which was partially attenuated after APC knockdown (Figure 6C). TUNEL staining and flow cytometry analyses showed that silencing YTHDF3 induced apoptosis in AGS cells, and silencing APC partially eliminated the effect of silencing YTHDF3 (Figure 6D,E). Therefore, silencing APC reversed the remission effect of silencing YTHDF3 on AGS cell malignancy.

Figure 6Silencing APC reversed the promoting effect of silencing YTHDF3 on AGS cells. (A) Cell counting kit‐8 (CCK‐8) assay was performed in AGS cells at days 1, 2, 3, 4, and 5. (B) The proliferation of AGS cells in the indicator group was assessed by 5‐ethynyl‐2′‐deoxyuridine (EdU) assay. (C) Transwell invasion assay in different transfection groups: si‐NC + si‐NC, si‐YTHDF3 + si‐NC, si‐NC + si‐APC, or si‐YTHDF3 + si‐APC. (D) Terminal deoxynucleotidyl transferase‐mediated dUTP nick end labeling (TUNEL) staining was used to detect the apoptosis of AGS cells in different transfection groups. Arrowheads indicate representative TUNEL‐positive cells. (E) Apoptosis was assessed in AGS cells from the indicated groups by flow cytometry with annexin V‐FITC/propidium iodide (PI) staining.  ^∗∗,∗∗∗^ *p* < 0.01, 0.001 vs. si‐NC + si‐NC.  ^###^ *p* < 0.001 vs. si‐YTHDF3 + si‐NC.

**Figure:**
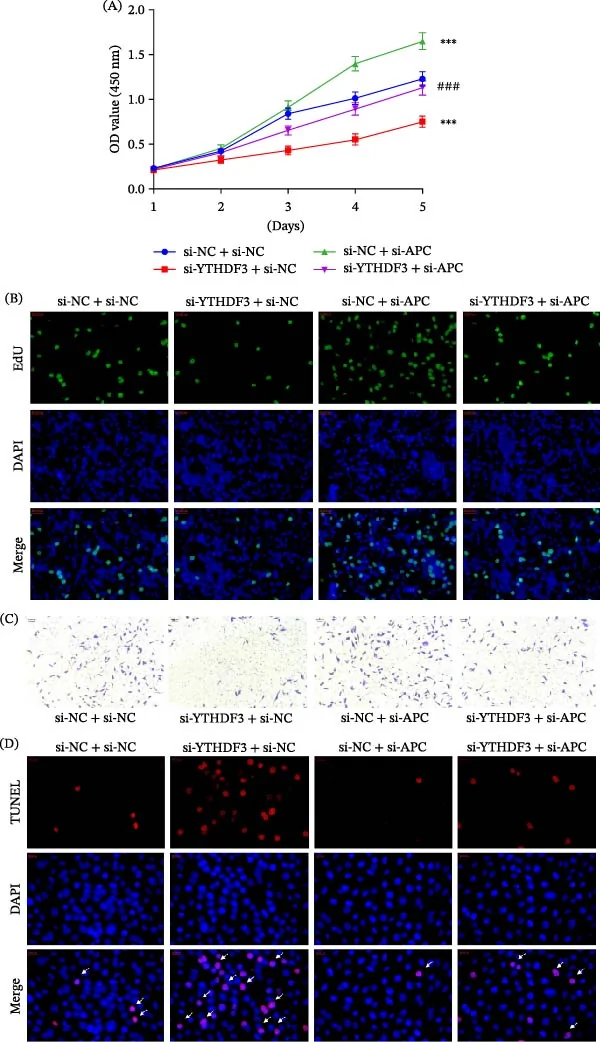

**Figure:**
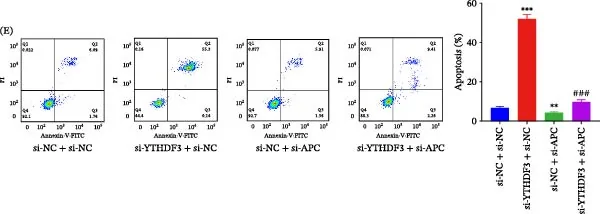

## 4. Discussion

STAD is common in East Asia and developing regions [27, 30]. Despite significant advancements in diagnosis and treatment, the recurrence rate and mortality rate of STAD have not been significantly reduced [31]. The persistently high recurrence and mortality rates have made the research and treatment of STAD a critical focus in the medical field. Consequently, discovering new regulatory molecules and potential therapeutic targets is essential to develop new therapeutic approaches for the benefit of STAD patients. We discovered that YTHDF3 facilitated the proliferation and invasion of STAD cells, and YTHDF3 could inhibit the stability of APC mRNA in an m^6^A‐dependent manner.

m^6^A, the most abundant RNA modification, plays key roles in various cancers [32]. YTHDF3 is an important m^6^A reader that has been associated with the progression of various malignancies, including breast [33] and colorectal cancer [34]. Previous studies have shown that YTHDF3 was linked to clinical outcomes, specific RNA signatures, and the immunosuppressive environment in STAD [17]. However, data on the mechanism of action of YTHDF3 in STAD are still limited. In the current study, the GEPIA database, Kaplan–Meier Plotter, and immunofluorescence were used to assess YTHDF3‐clinicopathological association. We found that the elevated YTHDF3 expression was closely associated with advanced T and N stages as well as distant metastasis. Additionally, elevated YTHDF3 expression levels were correlated with reduced survival rates in patients diagnosed with STAD. These findings indicated that YTHDF3 has the potential to serve as a valuable biomarker for prognostic assessment in patients with STAD.

To elucidate YTHDF3’s role in STAD, we performed in vitro assays. Silencing YTHDF3 notably diminished the proliferation and invasiveness of STAD cells, whereas YTHDF3 overexpression produced opposing effects. YTHDF3 has also been shown to promote the proliferation and migration of cancer cells, including glioblastoma [35] and hepatocellular carcinoma [36], which is consistent with our results. However, we only verified the effect of YTHDF3 on STAD in vitro and did not investigate its effect on STAD in vivo. Therefore, this study still has certain limitations.

The activation of proto‐oncogene mutations and the inactivation of tumor suppressors are important mechanisms of tumorigenesis, and m^6^A modification is widely found in genes related to this mechanism [37]. APC is a key tumor suppressor [38]. Moreover, APC expression was inhibited by METTL3‐dependent m^6^A upregulation on APC mRNA [39]. We found that YTHDF3 could bind APC mRNA, and RNA stability analysis showed that silencing YTHDF3 significantly promoted APC mRNA stability in AGS cells, while overexpression of YTHDF3 significantly impaired the stability of APC mRNA in SNU‐16 cells. In addition, DAA treatment and dual‐luciferase reporter assays further proved that YTHDF3 could reduce the stability of APC mRNA in an m^6^A‐dependent manner. We first show that APC is a direct m^6^A target of YTHDF3. YTHDF3, together with YTHDF1/2, regulates mRNA translation and decay [40]. Members of the YTH protein family derive their designation from the YTH domain, which is integral to the developmental processes and evolutionary progression of various organisms [41]. YTHDF2 could regulate the degradation of RNA, and m^6^A modification could occur in the 5′ UTR region of mRNA after heat shock stress [42] and could also induce the degradation of m^6^A‐modified APC mRNA [26]. Therefore, we speculated that m^6^A modification of APC may be involved in YTHDF1, YTHDF2, and YTHDF3. Nevertheless, a more in‐depth investigation into the specific mechanism is required.

To substantiate the impact of the YTHDF3/APC axis on STAD, additional functional rescue experiments were executed. It was observed that the reduction in cell migration and invasion capacities, along with increased apoptosis triggered by YTHDF3 silencing, could be partially restored by cotransfection with si‐APC. The Wnt/β‐catenin signaling pathway was involved in the proliferation of cancer cells, abnormal differentiation, enhanced migration and invasion, maintained the characteristics of tumor stem cells, and played an anti‐apoptotic role in cancer primarily through its abnormal activation [43]. Research has established that APC suppresses β‐catenin overactivation [44] and functions as a key negative regulator of the Wnt/β‐catenin signaling pathway [45]. In the canonical Wnt pathway, the β‐catenin stability is regulated by a destruction complex formed by axin, APC, casein kinase 1, and glycogen synthase kinase‐3beta (GSK‐3β) that phosphorylates β‐catenin at its N‐terminus and leads to its ubiquitylation and subsequent proteasomal degradation [46]. Loss or downregulation of APC impairs this process, resulting in reduced phosphorylation, causing β‐catenin nuclear accumulation [47, 48]. Our results are consistent with this. Specifically, silencing YTHDF3 increased β‐catenin phosphorylation and reduced both total and nuclear β‐catenin levels—effects partially reversed by cotransfection with si‐APC. In addition, YTHDF3‐knockdown suppressed Wnt/β‐catenin transcriptional activity and downregulated the expression of downstream targets c‐MYC and Cyclin D1, both of which were attenuated upon APC cosilencing. Therefore, our data suggest that APC, as a new target for YTHDF3, regulated the progression of STAD via the Wnt/β‐catenin axis, and these results may represent a new therapeutic target for STAD therapy.

However, this study also has limitations. In the clinical analysis, we used a paired *t*‐test to compare tumor and adjacent normal tissues, which controls for individual baseline differences but does not adjust for other confounders such as tumor stage or grade. In the in vitro experiments, confounders were controlled by experimental design rather than statistical adjustment. Future larger cohort studies with multivariate models are needed to confirm our findings. It is important to note that gastric adenocarcinoma encompasses several pathological subtypes, such as those defined by the Lauren classification (intestinal, diffuse, and mixed). These subtypes may exhibit distinct molecular profiles and clinical behaviors. Our current study investigated the role of YTHDF3 in STAD without stratification based on these subtypes, utilizing a mix of established cell lines and unselected patient tissues. While our findings demonstrate a significant protumorigenic function of YTHDF3 in the general STAD context, future studies are warranted to explore whether the YTHDF3‐APC regulatory axis exhibits differential activity or clinical relevance across specific pathological subtypes. Such investigations could provide more personalized insights into the prognostic and therapeutic implications of m^6^A modification in gastric cancer.

## 5. Conclusion

In summary, our investigation demonstrated that YTHDF3 correlated with adverse outcomes in STAD patients, and YTHDF3 promoted the migration and invasion of STAD cells while inhibiting their apoptosis. APC was identified as a pivotal downstream mediator of YTHDF3, whose activity was regulated by YTHDF3 through destabilizing APC mRNA in an m^6^A‐dependent manner. Together, these findings identified the carcinogenic role of YTHDF3 as an m^6^A reader and highlighted the therapeutic promise of manipulating the APC/β‐catenin signaling axis in STAD.

## Author Contributions

Yaqin He designed the study. Zijian Liu and Ruyi Li performed the experiments. Cao Zhang and Jun Wen wrote the manuscript.

## Funding

This work was supported by the “Study on the application of adenomatous polyposis coli gene mutations and circular RNA expression abnormalities in risk prediction and early diagnosis of gastric cancer.”(Grant 2021BEG03085) and the Ningxia Natural Science Foundation, entitled “Mechanism study of LINC01433 regulating E2F1 transcription via the let‐7a‐5p/YAP axis to mediate glycolytic reprogramming and promote gastric cancer metastasis.” (Grant 2024AAC03697).

## Disclosure

All authors read and approved the final manuscript.

## Ethics Statement

This study was approved by the Ethics Committee of the General Hospital of Ningxia Medical University.

## Consent

All participants signed informed consent forms.

## Conflicts of Interest

The authors declare no conflicts of interest.

## Data Availability

The data that support the findings of this study are available from the corresponding author upon reasonable request.
